# Supplementary material for: PPI-hotspotID for detecting protein–protein interaction hot spots from the free protein structure
Source: eLife. 2024 Sep 16;13:RP96643. doi: 10.7554/eLife.96643 (PMC11405013; doi:10.7554/eLife.96643)
Supplement: Figure 2—source data 1. [file elife-96643-fig2-data1.docx]

**Uncropped immunoblot images of the entire membranes for Figure 2b**.

**Uncropped images of the entire membranes for Figure 2c**.

**Phosphoimager file for Figure 2d**.

**
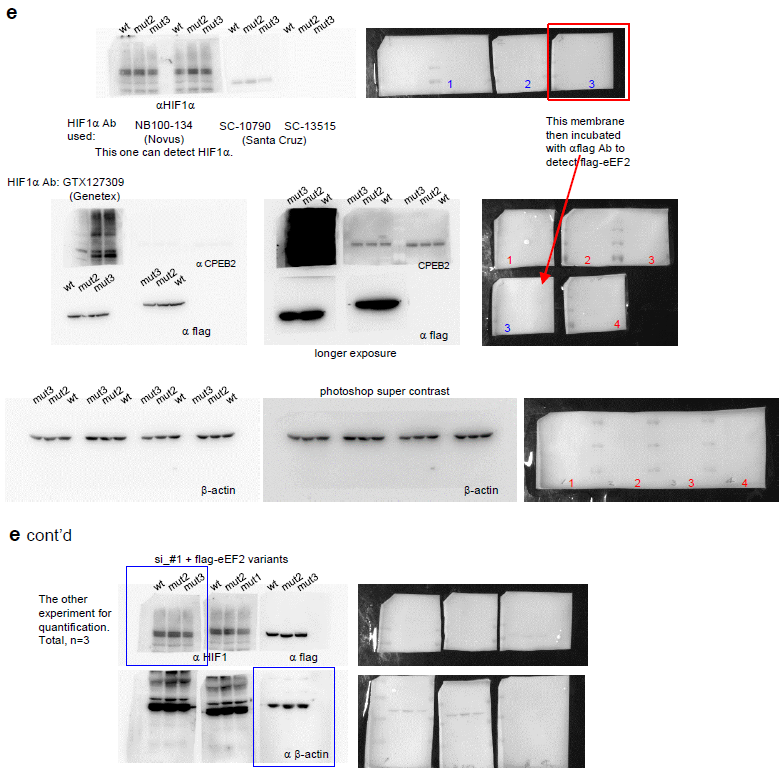
**

**Uncropped immunoblot images of the cut membranes for Figure 2e**.
