# Supplementary figures and images for: PPI-hotspotID for detecting protein–protein interaction hot spots from the free protein structure
Source: eLife. 2024 Sep 16;13:RP96643. doi: 10.7554/eLife.96643 (PMC11405013; doi:10.7554/eLife.96643)

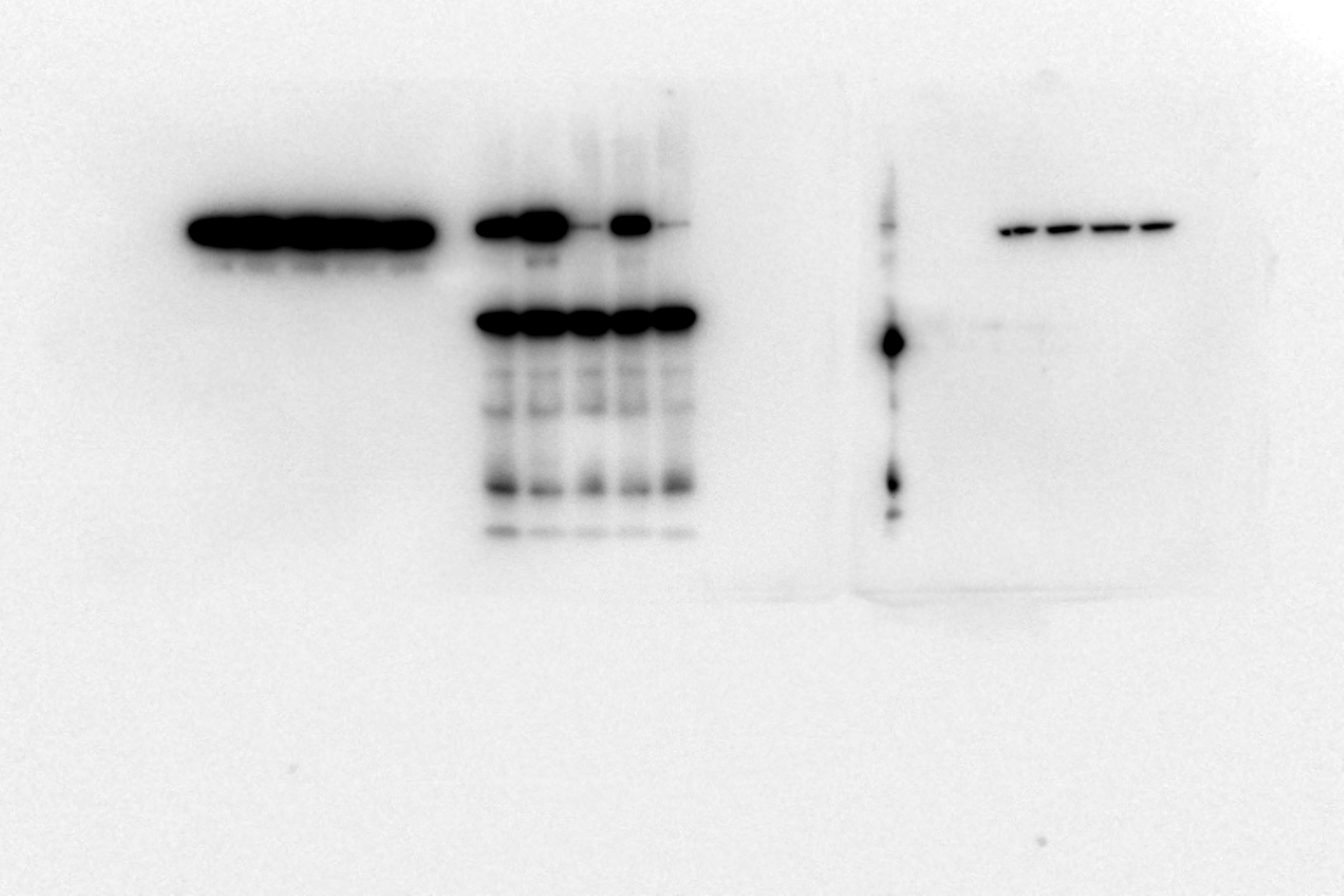

Supplement: Figure 2—source data 2. [file elife-96643-fig2-data2.zip › Source_Data_Fig2/co_IPeEF2_16.tif]

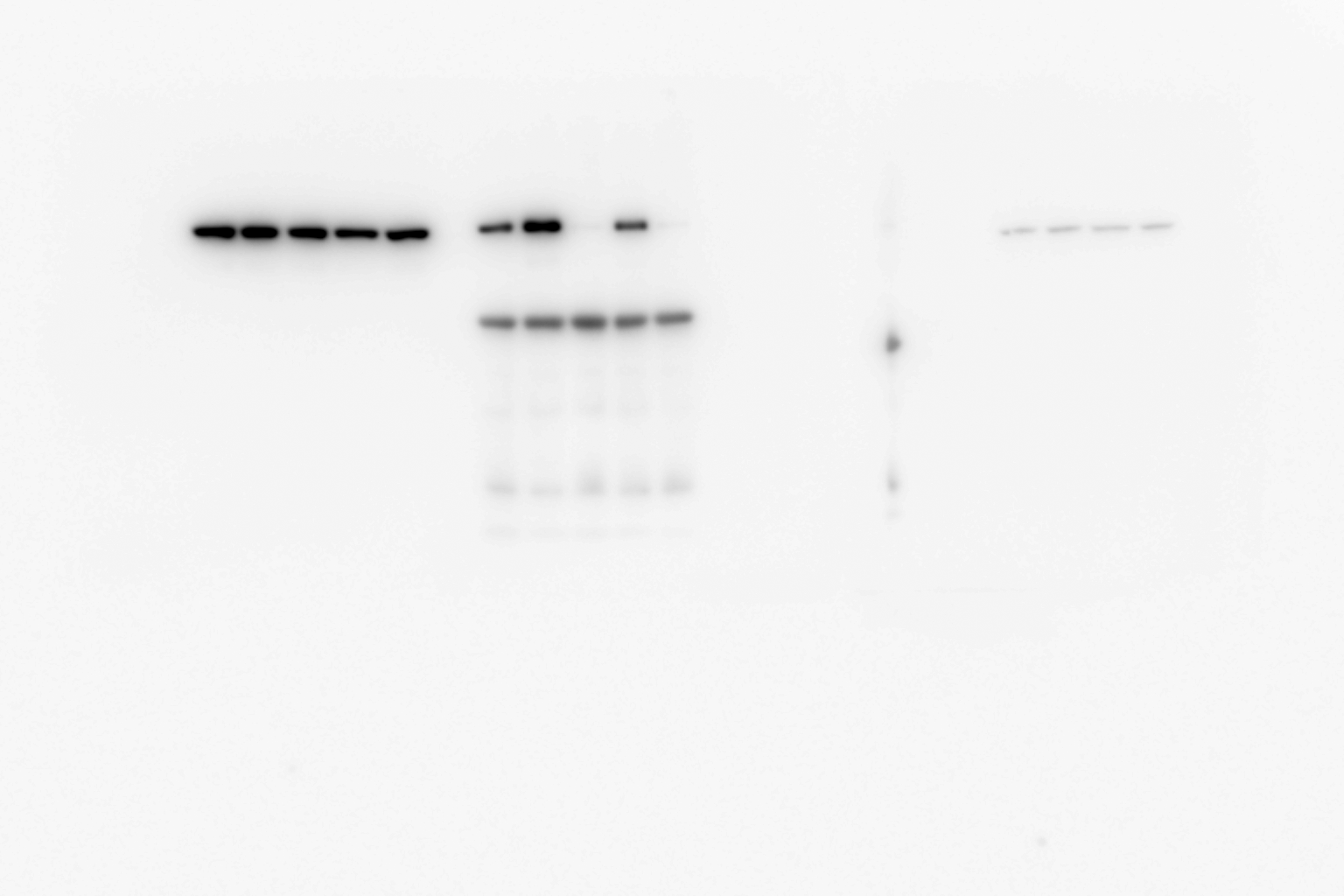

Supplement: Figure 2—source data 2. [file elife-96643-fig2-data2.zip › Source_Data_Fig2/co_IPeEF2_3.tif]

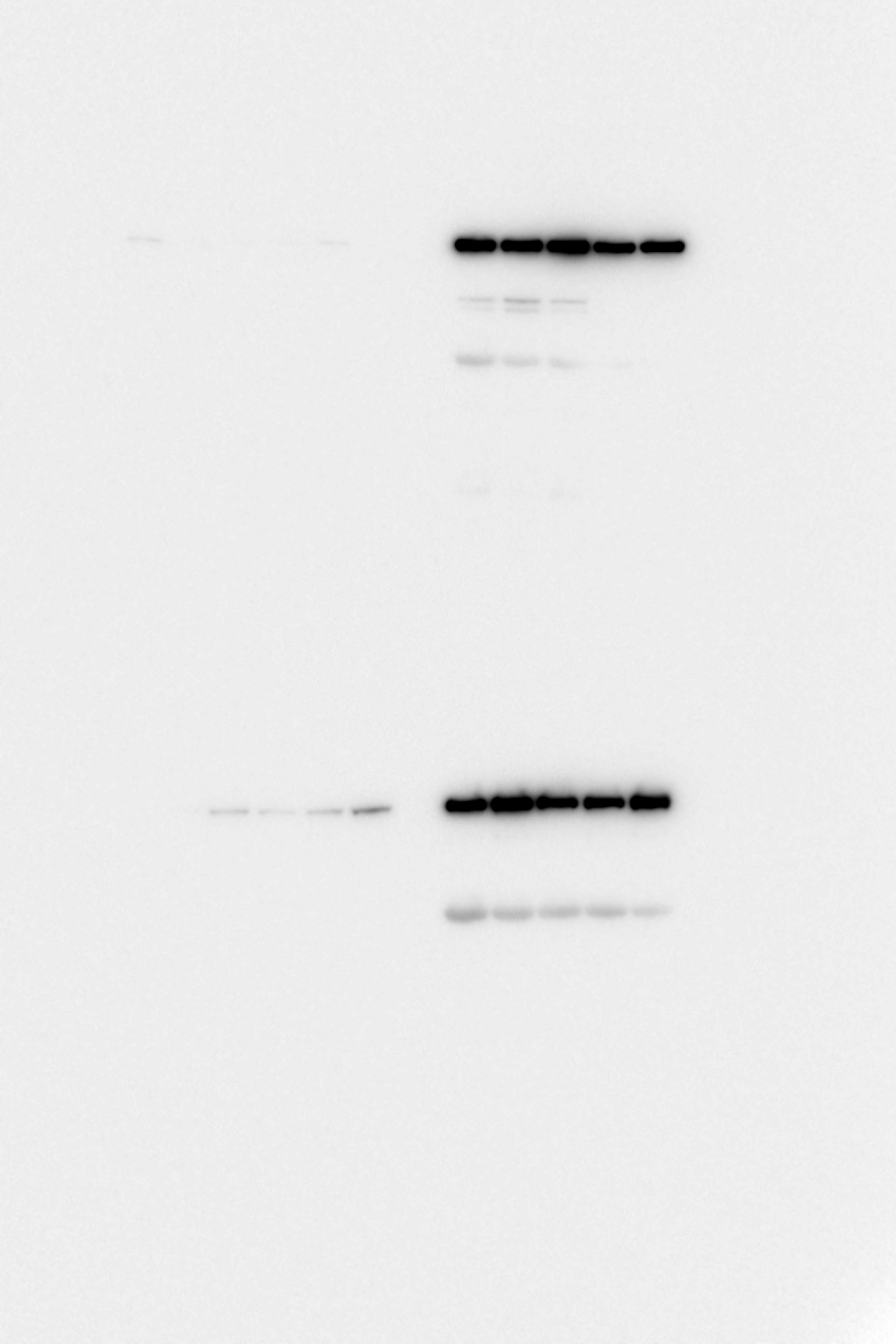

Supplement: Figure 2—source data 2. [file elife-96643-fig2-data2.zip › Source_Data_Fig2/eEF2_CP2 IP_epi_7.tif]

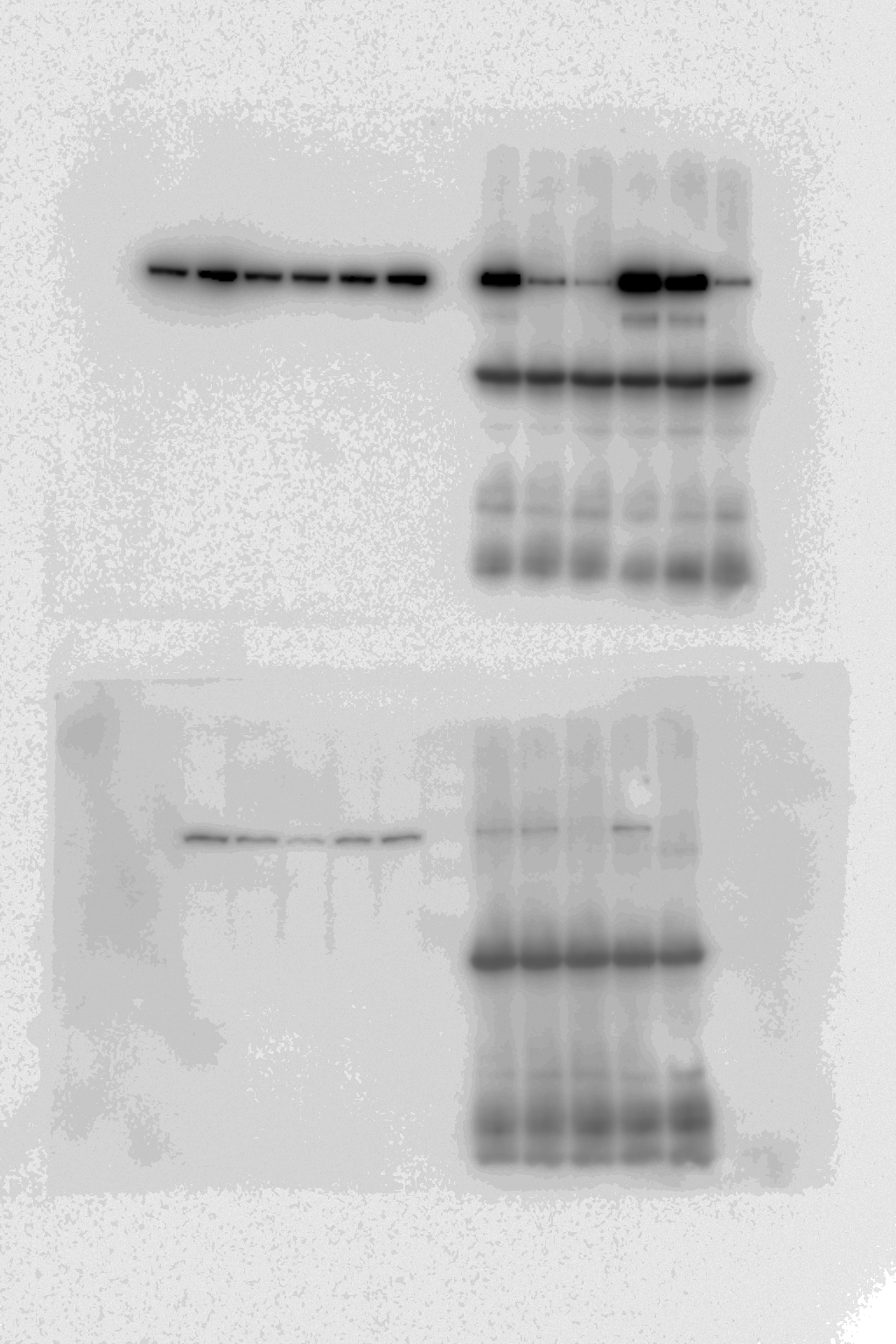

Supplement: Figure 2—source data 2. [file elife-96643-fig2-data2.zip › Source_Data_Fig2/eEF2_CP2coIP_9contrast.tif]

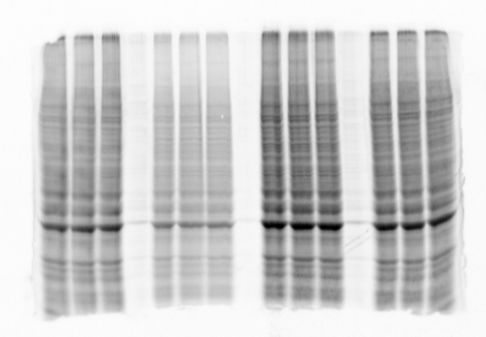

Supplement: Figure 2—source data 2. [file elife-96643-fig2-data2.zip › Source_Data_Fig2/S35Met061815.tif]
