# Supplementary material for: PPI-hotspotID for detecting protein–protein interaction hot spots from the free protein structure
Source: eLife. 2024 Sep 16;13:RP96643. doi: 10.7554/eLife.96643 (PMC11405013; doi:10.7554/eLife.96643)
Supplement: Figure 2—source data 3. [file elife-96643-fig2-data3.docx]

**Figure 2a – source data 1. Primer sequences.** The sequences of sense (forward) and antisense (reverse) primers used for site- directed mutagenesis.

### ^^
